# Living through conflict and post-conflict: experiences of health workers in northern Uganda and lessons for people-centred health systems

**DOI:** 10.1093/heapol/czu022

**Published:** 2014-09-11

**Authors:** Justine Namakula, Sophie Witter

**Affiliations:** ^1^ReBUILD Project, School of Public Health, Makerere University, Kampala, Uganda and ^2^ReBUILD Project, Queen Margaret University Edinburgh, Musselburgh, Edinburgh EH21 6UU, UK

**Keywords:** Health workers, conflict, post-conflict, retention, motivation, participatory research

## Abstract

Providing people-centred health systems—or any systems at all—requires specific measures to protect and retain healthcare workers during and after the conflict. This is particularly important when health staff are themselves the target of violence and abduction, as is often the case. This article presents the perspective of health workers who lived through conflict in four districts of northern Uganda—Pader, Gulu, Amuru, and Kitgum. These contained more than 90% of the people displaced by the decades of conflict, which ended in 2006. The article is based on 26 in-depth interviews, using a life history approach. This participatory tool encouraged participants to record key events and decisions in their lives, and to explore areas such as their decision to become a health worker, their employment history, and their experiences of conflict and coping strategies. These were analyzed thematically to develop an understanding of how to protect and retain staff in these challenging contexts. During the conflict, many health workers lost their lives or witnessed the death of their friends and colleagues. They also experienced abduction, ambush and injury. Other challenges included disconnection from social and professional support systems, displacement, limited supplies and equipment, increased workload and long working days and lack of pay. Health workers were not passive in the face of these challenges, however. They adopted a range of safety measures, such as mingling with community members, sleeping in the bush, and frequent change of sleeping place, in addition to psychological and practical coping strategies. Understanding their motivation and their views provides an important insight how to maintain staffing and so to continue to offer essential health care during difficult times and in marginalized areas.

**KEY MESSAGES**

- People-centred health systems have to start with listening to staff, especially those who provide the backbone of services on the ground. Life histories can be a powerful participatory research tool to elicit health worker experiences, views and motivation.
- Health workers’ coping strategies in this context were impressive. Personal faith, commitment to community and sheer fatalism were amongst their psychological defences. Many took pride in their inventiveness in managing in difficult conditions and evading rebels.
- The findings suggest the importance of selecting and favouring those with higher intrinsic motivation, especially in difficult times, when formal structures of promotion and recognition cannot function well, when pay is low and erratic and when working conditions are hard. During the conflict in northern Uganda, some health workers were equipped with and demonstrated values like empathy, professionalism and selflessness. This is an opportunity that can be seized after the conflict. Local employment, gender-sensitive policies, entry routes for people from poorer households and development of strong community links are all likely to be effective in attracting staff that will stay.
- The research also highlights the need for specific policies to protect health staff during conflict and to recognize and reward them for continued service in dangerous conditions.

## Introduction

This study is part of the ReBUILD health worker incentives research project.^1^ The project aims to understand the evolution of incentives for health workers post-conflict, and to derive policy recommendations for improving retention in those areas (Witter *et al.* 2011). The challenges in relation to recruitment, retention, distribution and management of health workers are recognized worldwide, but these can be exacerbated in conflict-affected areas (Macrae *et al.* 1996; Pavignani and Colombo 2001; Pavignani 2003; Witter *et al.* 2011). Although there is a legitimate focus on re-establishing services after conflict, the perspective of those who provide those services is often overlooked. It is an important part of responsive health systems that they are able to listen to and recognize the contribution of staff who have undergone trauma during conflict and yet, in many cases, continued to serve during and after that conflict. Understanding their perspective can contribute to improved policies and more effective healthcare services. This is an important challenge as more than 1.5 billion people live in countries affected by violent conflict. According to World Bank estimates, fragile and conflict-affected countries account for half of child deaths and will contain around a third of the world’s poor by 2015.^2^

Previous literature on conflict and fragile states has tended to focus on the effects of conflict on the health system in general. Documented effects have included human and capital flight, infrastructure destruction, and increased mortality and morbidity (Ohiorhenuan and Steward 2008). Country case studies on human resources for health for Cambodia, Mozambique, Liberia and Rwanda have indicated that health workers were targets for violence (Pavignani 2003). However, there is limited participatory research or research which seeks to understand why health workers stay in conflict-affected areas and how they cope.

For 20 years (1986–2006), the Acholi sub-region in northern Uganda experienced violent conflict as a result of fighting between the government and the Lord’s Resistance Army (LRA). This conflict claimed a lot of lives and displaced many people from their homes, besides devastating the social services and physical infrastructure in the region (Kindi 2010; Rowley *et al.* 2006; World Vision 2009). In some districts, such as Pader and Kitgum, displacement was massive, accounting for 95% and 90%, respectively, of the district populations (MoFPED 2002). The displaced populations were forced to seek refuge in internally displaced camps (World Vision 2009). Following the peace negotiations in June 2006, a period of tranquillity began (ARC 2007; Kindi 2010). The displaced population in camps was shifted into a number of unplanned satellite/transit camps. By 2010, more than 85% of internally displaced persons living in camps had returned to their villages of origin (USAID 2010) or moved to transit/satellite camps closer to their homes. However, some populations continued living in camps (Namakula *et al.* 2011). Some of the reasons for the slow return process included worries about the presence of landmines and unexploded ordinance, presence of bandits, the lack of services and limited access to their former villages (NRC and IDMC 2010; Namakula *et al.* 2011).

During the conflict, the health system in northern Uganda was divided into two: on the one hand, there was a functional camp-based health system run by international agencies and non-governmental organizations (NGOs). The government health service delivery was confined largely to towns where referral hospitals are located (WHO 2006). Post-conflict, movement of population from camps to original villages raised issues of access and equity as well as the need for health system reconstruction. Reconstruction efforts—including of health services—are now underway, with 1 billion dollars pledged by government and donors since 2006 to contribute to the peace and recovery development plan (PRDP 2007). There is a growing participation of the private sector (though with an urban bias). In terms of human resources for health, there has been a decline in expatriate health workers, with a few remaining in advisory or training posts, and a concern that projects may not be sustainable due to diminishing funds. It continues to be difficult to attract health workers to public health facilities in rural areas of the region (MOH *et al.* 2012).

This article presents a case study from northern Uganda, Acholi sub-region, where a participatory life history approach was used to understand the experiences and perceptions of health workers who had lived and worked through conflict in the region. It presents their experiences of conflict, how they coped, and what motivates them to stay in service, as well as recommendations for retaining staff in future. The concept of people-centred systems implies health care that is responsive to individual differences, cultural diversity and the preferences of people receiving care, which enables the patient to take as much control as possible over their own care, and which places the highest importance on individual dignity, respect and shared humanity (Robinson *et al.* 2008; Victorian Department of Health services 2006; WHO 2013). People-centred health systems therefore need staff who are not only adequate in number and competence, but also motivated and provided with an enabling environment to provide sensitive care (Victorian Department of Human Services 2006; WHO 2013). This is a major challenge for many countries even in peacetime, but presents particular difficulties in times of conflict and in the post-conflict period. The challenge is to understand what enabled some people to stay in service during the period of conflict to be able, where possible, to support those factors in other conflict situations, where these might be absent. In addition, one can learn from the resilience of health workers during the conflict period to better retain them in the post-conflict period. Not only will this knowledge help in other conflict and post-conflict situations, but also in peaceful countries that are experiencing difficulties in retaining staff, and thus failing at the first hurdle of the provision of people-centred health systems.

## Research methods

### Site and sample selection

Twenty-six life history interviews were conducted with health workers from Pader, Gulu, Amuru and Kitgum—as these were most affected by the LRA conflict, and contained more than 90% of the displaced people. Participant selection was purposive, based on district, sector [public and private not-for-profit (PNFP)] and number of years spent working in the Acholi region. The rationale for choosing health workers who had spent at least 10 years in the region was to enable us understand how their lives have changed since the war—a subject that could be discussed only by health workers with longer experiences in the region.

Although the study had aimed at interviewing equal numbers by gender, sector, level of health facility and district, the final distribution varied (Table 1), as few staff were found working at health centre II levels who had been in the region for 10 years or more. The distribution of interviews by district was seven for three of the districts and five for Amuru. Nineteen of the participants were female and seven male. The distribution across sectors was 17 in the public sector and 9 PNFP. These distributions reflect the pattern of staffing at facility level in this region. Two additional interviews were included with district officials who had worked in the region for the requisite period.

**Table 1:** Summary of participant characteristics

	Average
Age	42 years average (range: 30–60 years)
Time spent working in region	17 years average (range: 7–38 years)
Sex	23% M; 77% F
Cadres	Clinical officers (15.38%); nurses (57.68%); nursing assistants (7.69%); midwives (11.53%); others (7.68)
Districts	27% Pader; 27% Kitgum; 19% Amuru; 31% Gulu
Sectors	65% Public; 35% PNFP
Level of facility	Hospitals (31%); HC IV (15%); HC III and II (46%); others (8%)
Highest level of education (formal)	69% O’ level; 12% A’ level; 15% Diploma; 4% Degree

### Data collection

The process of tool development was a participatory one between team members from Uganda and the UK. A generic topic guide was produced by the UK lead researcher and was then adapted by the local team during training and pretesting. Field work was conducted in October 2012.

During the interview, a horizontal line was drawn on a piece of paper and participants were encouraged to record key events and decisions in their lives along the line. In this study, the lifeline was used to probe the decision to become a health worker, training experiences, their employment history, why they had stayed or moved between jobs, their job satisfaction, experience of conflict, coping strategies during and after conflict, and experience of different policies. The life line was used as a tool to guide discussion (as a useful prompt and cross-checking source during the interview) and emerged as a visual representation/summary of key events, as perceived by the participant at the end of the interview.

The potential strength of this tool is that it permits a holistic analysis of health workers’ lives, taking into account personal and contextual factors. Although life histories have been used in the health sector for research into patient groups (Cole and Knowles 2001; Miller 1994), the nursing profession (Cole and Knowles 2001; Leininger 1985) as well as poverty and well-being (Bird 2008), it has not been used, to our knowledge, to probe the perceptions of health workers in low- and middle-income settings before.

### Ethical approval

Ethical approval to conduct the study was granted by Makerere University School of Public Health Higher Degrees Research and Ethics Committee; the Uganda National Council for Science and Technology and the University of Liverpool in 2012.

### Data analysis

Data analysis was guided by the framework analysis approach of Ritchie and Spencer (1994) and analysis framework stages provided by Richie and Lewis (2003) and ATLAS TI software version 5.0 as described later. The analysis framework involved the following stages: familiarization, listening to the audio recordings, reading field notes, coding and identification of key themes, merging themes, searching for key findings under each theme, comparing and finding associations, and provision of explanations/meaning (Ritchie and Lewis 2003).

Audio tapes were listened to and compared with notes taken during interviews to fill in the gaps in information that could have been left out or miss-recorded during note taking. Audio recordings were transcribed verbatim so that original meanings are not lost. Transcripts were read several times to get an overall picture and then recurring preliminary themes were identified and used to create codes (see Supplementary File for a summary of the coding tree).

A code book was generated and agreed upon between team members in Uganda and in the UK. Data were then prepared for entry into ATLAS Ti by filing transcripts using identifiers such as district, ownership, type of facility, cadre and gender. Filed transcripts and codes then uploaded into ATLAS TI Software version 5.0 and coding nodes were attached to quotations in each. ATLAS query reports were generated and printed out for each code and further familiarized to identify more sub-themes. Similar sub-themes were merged together to create themes, whereas in some cases sub-themes were created. Finally, quotations that epitomized the emerging themes were identifies and agreed upon by the research team.

### Study limitations

The main limitation to be noted is that by selecting those who had worked in the region for 10 years or more, this study effectively adopts a ‘positive deviance’ approach: it cannot illuminate the situation or choices of health workers who left, but can provide in-depth insights into those who stayed. It is also based on a cohort of workers who experienced the conflict—how their experiences map on to those of younger workers now joining the workforce is a matter for further investigation. As with all research, the translation of the findings of this study to other settings has to be done sensitively, with careful consideration of contextual differences.

## Results

### Experiences of conflict

The effects of the LRA-generated conflict can be categorized into two: effects on health workers’ health and security, and effects on health workers’ working conditions. Effects on health workers’ security and health included abduction, ambush, injury and death. Conflict-related challenges in relation to the health worker’s working conditions included disconnection from social and professional support systems, displacement, limited supplies and equipment, increased workload and long working days, and lack of pay. Breakdown in transportation and dangerous roads led to their physical, professional and social isolation.

During the conflict many health workers lost their lives or witnessed the death of their friends and colleagues. Those who survive death remained traumatized and in constant fear for their lives.

*“I lost so many friends; we lost even some of our staff during the ambushes on our way to Gulu” (Female participant, Kitgum)*

*“Then you could hear gunshots, someone shooting just very near at times you feel like you are going to be shot at that time, that fear was there” (Male participant, Amuru)*

Participants noted that as health workers, they were a particular target for abduction, as the rebels needed medical attention. Almost all respondents witnessed traumatic events, being abducted themselves or witnessing the abduction or near-abduction of colleagues. Many of the participants who were abducted were lucky to have been set free after a period of time which ranged from hours to days and months.

“*In 1986, I was abducted by rebels for 2 days because they expected me to help them. They said, ‘this is the right man to help us in the bush’ [...] but a rebel who was born in Mucwini, where I also come from saved me and released me and then I found my way back home in Mucwini*’’ *(Male participant, Kitgum)*

Health workers experienced a number of ambushes by rebels and they had vivid memories of them. Ambushes were common along the roads connecting districts or at particular road junctions. Many of them survived death or got lasting physical injuries.

“*In 1999 after I qualified, I went to Kitgum to do an interview. We were five clinical officers from Pader. On our way back, we were ambushed, one of our colleagues was shot in the chest but he survived narrowly. The rest of us survived as well” (Male participant, Pader)*

Infrequent travel due to fear of ambushes and landmines created disconnection from professional support, medical supplies and equipment and from monthly salaries, which were often paid in the other districts. Although in some cases security was provided in terms of convoys to escort vehicles with supplies and patients, health facilities in remote areas remained disconnected from supplies. Health workers in such areas had to endure shortages, to innovate, or lobby for some buffer supplies from NGOs. Where there was no security from the army, health workers were forced to travel to other districts at risk to their lives.

“Yeah I walked from there up to the health centre where I had been posted because the vehicle that was taking me was stopped by the army men, not to continue ahead because of the insecurity, landmines had been planted on the way” (Female participant, Gulu)

During the conflict, there was limited supply of medical equipment, as a result of raids by rebels, and inability to move to bring in new stock from the other districts due to the insecurity. In addition, the overwhelming number of casualties during the time rendered the available supplies inadequate.

“*Yes they even went with some vaccines, they went with drugs, they went with very many things” (Female participant, Gulu)*

*“In Adilang, [...] I remember struggling to help a woman kneeling with no bed but just on the floor so that was the worst experience I had. I was also pregnant and I got a miscarriage” (Female participant, Pader)*

The conflict also resulted in increased work load and long working days for those health workers who worked in facilities that remained functional during the war (mainly the private not for profit facilities and those facilities located in camp settlements). War casualties, high rates of illnesses and epidemics such as cholera (2000–2001) and Ebola (2005–2006) all contributed. As a result, health workers were emotionally and physically affected.

“The number of clients overwhelmed us because we could get about 700 to 800 cases. You could be on night duty but are required to report at 7.30 every morning. During that one year I lost weight and became very thin because the work was too hectic” (Female participant, Kitgum)

As a result of conflict many health workers fled to safer areas within the same districts, usually urban areas, or to other areas within or outside Acholi sub-region. In many cases, they moved to facilities elsewhere or abandoned health work for some time.

### Coping strategies

In light of all the above challenges, health workers were not docile; they actively created coping strategies to deal with the context. These included practical safety measures, such as mingling with community members, sleeping in the bush, frequent change of sleeping place or staying stationary. To mingle with patients, they underlined the need to build trusting relationships with the community. At this point, their uniforms changed from being a symbol of smartness to a cause of vulnerability to abductions by the rebels.

“Other staff who feared staying in the nurses quarters again could run and sleep with the patients in the hospital because when you are abducted as a nurse you will not escape! So at least when the rebels find you among the patients they will leave you thinking you are also a patient and at night we could not put on uniform so that you are not detected” (Female participant, Kitgum)

Localized conflict such as cattle rusting from Karimojong warriors also created insecurity for some of the respondents. One responded by befriending some soldiers, doing military training, acquiring a gun and becoming a fighter, in addition to his health work.

In addition to practical measures, participants reported important psychological coping strategies, including getting support from their managers, elders and communities, getting strength from their faith and from a sense of service to the community, being fatalistic about their situation, and, conversely, taking pride in their resilience and inventiveness in adverse circumstances.

“As a human being you have to persevere with the pain because there was nothing I could do because these people could come any time they want. We would just leave all those problems to God. Nothing as a human being you do apart from praying to God” (Male participant, Kitgum)

*“If we were to run away who would now help them? So we persisted and slowly the fear disappeared” (Female participant, Amuru)*

*“[...] we would work until our gloves were over, because of too many injuries and at times we would use kavera [polythene bags], because you cannot leave a person to die, so we used ‘kavera’” (Male participant, Pader)*

“We just continued surviving like that. One good thing that I saw was health workers became committed and I remember there was a strike during that time also, where the health workers were striking and did not want to work, but for us we continued working. Prior to the strike day, we sat down and said ‘our people have suffered enough and we cannot go back, we cannot join them or the rest of the country in the strike’. Let’s remain and continue to work” (Male participant, Kitgum)

To cope with the increased workload, health workers had to take on more complex cases (higher levels of responsibility than those they were qualified to do) and work in shifts. They were motivated by a sense of vocation.

“[...] this is not my role, it was a role of a doctor, and you know it very well but we don’t want to bury the foetus in the dead mother’s womb - so I had to separate these people. I had to struggle with the knowledge I had” (Female participant, Pader)

“There was a time when there was an outbreak of meningitis and the nurses were helping a lot, because when somebody comes you just do a lumbar puncture, which we are not supposed to do, but you know there are settings when there are no doctors” (Female participant, Amuru)

Health workers also coped with the aid of external support and through income generation strategies, such as alcohol brewing and selling foodstuff*,* or relying on the community. External support came from the army, though its protection was only partially effective. In addition, NGOs and external donors, including missionaries, provided important practical and moral assistance during the conflict period. The support of expatriate doctors was also important in encouraging some to continue working in difficult conditions.

“*[...] Aah!, It was too much for us but only that [...] professor [an Italian expatriate] told us that ‘supposing you were the one who is, you put yourself as if you were the person?’ So that taught people to work [...]” (Male participant, Pader)*

“*[...] around 1986–87, there was no salary so I brewed alcohol to survive” (Female participant, Kitgum)*

### Motivators and de-motivators to stay in service

There were a number of factors which emerged as motivators (or de-motivators, if absent) for health staff, during and after the conflict. These included the following:

1. *Community support* and practical assistance of various sorts, provided by the district, external agencies and small gifts from patients.
  “In the first place I was motivated by community of Lira Kato, the present Apono sub-county. They were in total support of my well being, they were able to provide food for me. Another motivation was the district which was able to provide for me means of transport. UN High Commission for Refugees (UNHCR) gave us food and also good things like blankets and mattress because we had children” (Male participant, Pader)
2. *Appreciation* by supervisors and the community.
  “You know motivation is not physical things only and that particular in-charge could motivate even through thanking you when you have done some work” (Female participant, Amuru)
3. *Effective working conditions* such as having access to equipment, referral transport, among others.
  “One thing that I would like to see in place is having a facility with good settings with all the equipments, which is number one. I would want to have the outpatient department (OPD) fully equipped. That would make me happy. Another thing is get me accommodation and settle me down there. Then whenever am moving to the headquarters, I need a motorcycle to help me move. Then the last one which important but last is that I should have good co-ordination between the members of the community and the health centre” (Male participant, Pader)
4. Being able to *learn and develop one’s skills* and roles were important motivators for many, even those in relatively lowly posts. They were eager for further training and certificates to demonstrate their advancing skills.
  “I liked my job because of the experience I got while in the children’s ward [which] helped me gain a lot of experience and I knew a lot of things beyond my training” (Female participant, Kitgum)
  *“Because you are there and sometimes you may not be allowed to do some things. So you don't have time to learn [...] In fact I was not happy. The little knowledge I had, if I did not use it, it would disappear” (Male participant, Amuru)*
5. *Formal promotion* that corresponds with one’s qualification, and is reflected in a corresponding salary, was perceived as recognition of their contribution during the conflict and as a (missing) motivator that affects performance in the post-conflict period.
  “Up to now I have not been promoted I must tell you. I am earning a salary of an enrolled nurse! I qualified in 1998 and up to now no promotion. So I feel that burden and it has discouraged me from doing the best of my capacity” (Female participant, Kitgum)
6. *Employment benefits*, such as food, accommodation, transport, free health care, uniforms, and other occasional additions, such as sponsorships for their children.
  “I want to go to a place where the leader, the manager is good, a very conducive environment, within the working area and accommodation has to be there” (Female participant, Amuru)
7. *Good leadership and communication* in the workplace (staff encouraged to express themselves). Poor relationships with supervisors, lack of recognition and absentee managers were a cause of dissatisfaction, sometimes causing people to leave their posts.
  *“You should talk to the health workers and get their views. It should not be this push method, they should call them, have open discussion” (Female participant, Gulu)*
  “We were never given freedom of speech and whenever you talked something they would say you are not respectful; they never wanted anyone to talk the truth about them” (Female participant, Kitgum)
8. *Regular and adequate pay*, especially after the end of the war and for staff reaching the expensive time of life (with children at secondary school).
  “[...] also top up their salaries and also be able to recognize – because actually the nurses are never recognized in spite of the work that they do” (Female participant, Kitgum)
9. *Flexible working*—ability to augment salaries and build up some assets. The public sector tended to offer higher pay for many cadres (though not for all), with fewer restrictions on outside earning opportunities, and greater access to training opportunities and pension rights. Some participants also felt that formal employment policies, such as the right to paid leave, were more respected in the public, compared to the PFNP, sector.
  “If you‘re in government, you have a very big chance of going for workshop [...] there are very many scholarships [...] you know if you go higher you are going to do less work and you will be getting more money” (Male participant, Pader)

## Discussion

### Reflection on the methods

The life history tool was found to be an effective participatory approach to understand the embedded experiences of health workers in the Acholi sub-region. People-centred health systems have to start with listening to staff, especially those who provide the backbone of services on the ground. Some aspects which arose—such as motivation to join the profession, and how this affected subsequent career trajectories—are described elsewhere (Namakula *et al.* 2013). This article has focused on their experiences of conflict, how they coped and what motivated them to stay in service. As noted in the Methods section, by selecting health workers with more than 10 years of experience in the region, we were able to focus on a group of largely mid-level health cadres who stayed in service. Understanding their motivation and their views provides an important insight into how to maintain staffing during difficult times and in marginalized areas. Moreover, by taking a retrospective perspective over the period of their lifetime, we were able to understand how their personal history affects current motivation and retention.

### How the findings link to context

The majority of those interviewed were mid-level cadres, with an average time of 17 years spent working in the region. On one hand, this reflects the existing staffing mix, which is dominated by mid-level cadres, whereas on the other hand it is indicative of the difficulties of attraction and retention of more qualified cadres such as clinical officers, doctors, and medical officers in hard to reach areas, including those in conflict (Matsiko 2010; MOH 2006).

Personal factors, including gender, appear to have played an important role: the staffing of the facilities is predominantly female, which may reflect a number of factors, including role models of service by female relatives, the ties of family commitments in the area, greater resilience, and higher female attraction to the mid-level cadre roles. Gender roles were cited as a hindrance to upgrading, particularly for the female participants.

In relation to contextual factors, the long period of conflict and its aftermath was enormously significant for the respondents. Hardship, fear and direct or indirect casualties (injury to them or to those near to them) were reported by all respondents, who had nevertheless stayed in the areas.

Their recommendations, which link closely to factors which motivate and demotivate, are however not substantially different from those in non-conflict settings (Rourke 2010; UNFPA 2011; WHO 2010). They reflect a range of needs, from basic and material (adequate money to live on) to social (good relationships) and self-actualizing (the ability to be effective in your role and develop yourself). The main difference may be a greater sense of vocation—of serving God and the local community, which was demonstrated in their career history as well as their comments on motivation.

### Lessons arising and links to existing literature

In this context, some policy levers emerge as significant in boosting recruitment and retention. Recruiting from local areas is likely to be productive—these respondents tended to stay in their districts, and ties of family and land were part of their ‘stick’ factors. They were also loyal to the sector (and often facility) which first sponsored their training, suggesting that is also effective at retaining them. Offering training routes which favour those with lower levels of education also appears to be important, allowing incremental steps which may include volunteering, on the job training and access to in-service training so that those who have less access to education can nevertheless enter and progress. These people are more likely to be motivated to stay in hardship areas.

In general, the findings suggest the importance of selecting and favouring those with higher intrinsic motivation, especially in difficult times, when formal structures of promotion and recognition cannot function well, when pay is low and erratic and when working conditions are hard. During the conflict in northern Uganda, some health workers were equipped with and demonstrated values like empathy, professionalism and selflessness. This is an opportunity that can be seized after the conflict.

Our findings are consistent with those of a study by Serra *et al.* (2010) in Ethiopia which showed that intrinsic motivation may be developed right at the entry of the health profession and is propagated throughout one’s career. Such motivation is common among those who started their career in not for profit facilities. Those with intrinsic motivation were found to be satisfied even when they were getting lower salary than their colleagues (Serra *et al.* 2010).

This does not imply that remuneration and promotions should be neglected, however, for people in hardship areas. Pay is not the main motivator but matters, as does flexibility about other activities, assuming that pay remains low relative to living costs. Other benefits in kind are highly valued (and perhaps also reflect the recognition to which health workers aspire). As conflict wanes and as they advance in their career and face the most expensive phase of family life (having children at secondary school), mid-level workers require pay and other opportunities (such as training and promotions), which recognize their contribution. This matches existing literature on health worker motivation and retention (Hertzberg *et al.* 1959; Ssengooba and Rutebemberwa 2005).

One observation relates to the relationship between satisfaction and retention. Our respondents were asked about their satisfaction with their job at different points in their career and while satisfaction varied according to work, personal and contextual factors, low satisfaction had not led to a career change or even necessarily less willingness to work. For those who have strong internal motivation—‘I work for God and my country’ as one respondent put it—lower satisfaction may not cause lower effort or achievement. Some studies have found that satisfaction was a result of being able to handle health problems, acknowledgement by the community, and being able to make a change in the community (VSO 2011). In some literature it is argued that that there is no significant link between intrinsic and extrinsic satisfaction and retention, rather that satisfaction is generated as a result of career commitments and organizational commitment, which could also have a link to leadership strategies (Land 2003; Peace 1994). This was raised specifically for the not-for-profit sector.

## Conclusion and lessons

Our study adds to the existing literature on health workers in conflict by focusing on the experiences and motivation of a group of health workers who stayed during and after conflict. It provides details on their coping strategies and compares experiences across public and PNFP sectors. It uses health workers' voices and reflects their agency in these difficult circumstances. To our knowledge, it is the first time that life histories have been used to understand the situation of health workers in low- and middle-income countries.

The interviews raise questions on how best to protect health workers during conflicts. In some cases, health workers may be protected under an agreed policy of not disrupting services; however, in northern Uganda health workers were specifically targeted as being of use to the rebel forces. In this context, or in conflict-affected areas where this may occur, training in how to react (and agreed procedures with the local community) might be advisable. The trauma that health workers who stayed through conflict have undergone is also rarely recognized. Health services should recognize and celebrate the contribution of those who continued to serve on the front line during conflict-affected times. In times of conflict, alternative mechanisms for getting pay direct to workers should be developed. Insecurity means that opportunities to move or access services such as banks will very likely be limited.

More generally, and in the post-conflict period, the findings suggest that incentive policies need to target continued motivation and commitment of mid-level cadres because they are more likely to stay in these areas, while also putting in place programmes to upgrade their skills to be able to handle complications effectively. Although local employment can aid retention, the reverse of this coin may be discrimination against people born out of the area, particularly in a decentralized system. This should be controlled through sensitization of the local leaders and communities. Gender was an also an important factor, influencing issues such as joining the profession, upgrading, coping strategies and roles at work. Gender-sensitive policies are needed—for example, supporting women to be trained and promoted without compromising their wider roles in the household.

Human resource management policies should focus on maintaining the intrinsic motivation which many health workers bring when they join the profession through practices which foster good communication, support professional pride, and develop the links with the community—all of which are motivators to stay in service, especially in remote situations and in conflict affected areas.

## Supplementary Data

Supplementary data are available at *HEAPOL* online

Supplementary Data

## Endnotes

^1^ See: www.rebuildconsortium.com

^2^ http://web.worldbank.org/WBSITE/EXTERNAL/NEWS/0,,contentMDK:20042303∼menuPK:34480∼pagePK:64257043∼piPK:437376∼theSitePK:4607,00.html
