# Supplementary material for: Living through conflict and post-conflict: experiences of health workers in northern Uganda and lessons for people-centred health systems
Source: Health Policy Plan. 2014 Sep 11;29(Suppl 2):ii6–ii14. doi: 10.1093/heapol/czu022 (PMC4202915; doi:10.1093/heapol/czu022)
Supplement: Supplementary Data [file supp_29_suppl-2_ii6__index.html]

Living through conflict and post-conflict: experiences of health workers in northern Uganda and lessons for people-centred health systems — Supplementary Data 

# Living through conflict and post-conflict: experiences of health workers in northern Uganda and lessons for people-centred health systems

## Supplementary Data

files

**Files in this Data Supplement:**

- Supplementary Data - docx file
- Supplementary Data - docx file
