# Supplementary material for: Living through conflict and post-conflict: experiences of health workers in northern Uganda and lessons for people-centred health systems
Source: Health Policy Plan. 2014 Sep 11;29(Suppl 2):ii6–ii14. doi: 10.1093/heapol/czu022 (PMC4202915; doi:10.1093/heapol/czu022)
Supplement: Supplementary Data [file supp_czu022_Supplementary_file_Uganda_HWs_conflict-codes_for_analysis.docx]

**Burden**

Table 1: Examples of codes, condensed meaning units and interpretation, sub- themes and themes from content analysis of effects of conflict on health workers

| **Preliminary code for ATLAS Ti Soft ware** | **Condensed meaning unit**  **Description close to the text** | **Condensed meaning unit**  **Interpretation of the underlying meaning** | **Sub-theme** | **Theme** |
| --- | --- | --- | --- | --- |
| Major shocks during the war | Health workers were their target | Risk of abduction | Effect on Health worker’s health and security | Effects of the LRA on health workers/ Experience of conflict |
|  | Managed to escape | Risk of abduction |  |  |
|  | Abducted the incharge and killed the nursing aide | Abduction and  Death |  |  |
|  | Lost some of our staff during the ambushes on the way | Ambush and  Death |  |  |
|  | Was abducted by rebels for 2 days | Abduction |  |  |
|  | Were ambushed, one of our colleagues shot but in chest but survived, we survived as well | Ambush, Injury,  Narrow miss of death |  |  |
|  | Had to risk if you didn’t have money to go up to Kitgum | Infrequent travel  Risk of life  Disconnection from monthly salaries | Effects on conflict on health workers’ working conditions | Effects of the LRA on health workers/ Experience of conflict |
|  | Worked until our gloves were over | Increased workload due to war related injuries.  Limited supplies |  |  |
|  | Started working from 6.00pm to around 4am | Increased workload  Long working hours/days |  |  |

Table 2: Examples of codes, condensed meaning units and interpretation, subthemes, categories and themes from content analysis of narratives about coping strategies

| **Condensed meaning unit**  **Description close to the text** | **Condensed meaning unit Interpretation of the underlying meaning** | **Sub-theme(s)** | **Theme(categories)** | **Theme**  **(also code for ATLAS ti soft ware)** |
| --- | --- | --- | --- | --- |
| Not put on Uniforms | Uniform as a symbol of vulnerability to abductions | Mingling with patients | Coping strategies in relation to abduction | Coping strategies |
| went and slept in the bush | Change of sleeping place( from comfort of home) | Sleeping in the bush | Coping strategy in relation to abduction | Coping strategies |
| If you are stationed you wouldn’t bother so much | Staying stationary helped in avoiding ambushes | Staying stationary | Coping strategy in relation to ambush | Coping strategies |
| At times we would use Kavera [polythene bags] | Innovation in face of limited supplies  Risk to the health worker’s life(links to sub theme of working conditions) | Coping with limited supply | Coping with poor working conditions | Coping strategies |
| Not my role, it was a role of a doctor. | Taking on more complex cases than those they are qualified to do) | Task shifting | Coping with poor working conditions | Coping Strategies |
| No salary so I brewed alcohol to survive | Innovating an income generating activity to cope with absence of salary | Coping with disconnection from professional support | Coping with poor working conditions | Coping Strategy |

Table 3: Examples of codes, condensed meaning units and interpretation, subthemes, categories and themes from content analysis of narratives about motivators and demotivators during and after the conflict

| **Preliminary code for ATLAS Ti Soft ware** | **Condensed meaning unit**  **Description close to the text** | **Condensed meaning unit Interpretation of the underlying meaning** | **Sub-theme(s)** | **Theme** |
| --- | --- | --- | --- | --- |
| Job likes | Community gave me food | Community support | Community support | Motivators during the war |
| Job like | UNHCR gave us food, blankets, mattresses for our children | Support from external agencies | Practical assistance | Motivators during the war |
| Job dislikes | Our in-charge was rude | Disrespectful supervisor  Bad leadership | Poor relationships with supervisors | Demotivators/dissatisfiers |
| Job likes | Like a place with OPD fully equipped | Access to equipment, referral | Effective working conditions | Motivators post conflict  ( demotivators if absent) |
| Job likes | Top up our salaries |  | Regular and adequate pay | Motivators post conflict  ( demotivators if absent) |
| Job dislikes | Up to now have not been promoted | Promotion should correspond with qualification overtime | Formal promotion | Demotivators during and after the conflict |
