# Supplementary material for: Living through conflict and post-conflict: experiences of health workers in northern Uganda and lessons for people-centred health systems
Source: Health Policy Plan. 2014 Sep 11;29(Suppl 2):ii6–ii14. doi: 10.1093/heapol/czu022 (PMC4202915; doi:10.1093/heapol/czu022)
Supplement: Supplementary Data [file supp_czu022_Table_1.docx]

**Table 1: Summary of participant characteristics**

|  |  | Average |
| --- | --- | --- |
| 1 | Age | 42 years average (range: 30-60 years) |
| 2 | Time spent working in region | 17 years average (range: 7-38 years) |
| 3 | Sex | 23% M; 77% F |
| 4 | Cadres | Clinical officers (15.38%); Nurses (57.68%); Nursing assistants (7.69%) Midwives (11.53%); Others (7.68) |
| 5 | Districts | 27% Pader; 27% Kitgum; 19% Amuru; 31% Gulu |
| 6 | Sectors | 65% Public; 35% PNFP |
| 7 | Level of facility | Hospitals (31%); HC IV (15%); HC III and II (46%); others (8%) |
| 8 | Highest level of education (formal) | 69% O’ Level; 12% A’level; 15% Diploma; 4% Degree |
